# Expression profiles of circular RNAs in human colorectal cancer based on RNA deep sequencing

**DOI:** 10.1002/jcla.22952

**Published:** 2019-06-06

**Authors:** Jiaxin Ge, Yanping Jin, Xueyou Lv, Qi Liao, Cong Luo, Guoliang Ye, Xinjun Zhang

**Affiliations:** ^1^ Department of Gastroenterology, The Affiliated Hospital of Medical School Ningbo University Ningbo China; ^2^ Department of Biochemistry and Molecular Biology and Zhejiang Key Laboratory of Pathophysiology Ningbo University School of Medicine Ningbo China; ^3^ Department of Abdominal Oncology Zhejiang Cancer Hospital Hangzhou China

**Keywords:** circular RNA, colorectal cancer, hsa_circ_0142527, KLF4, RNA deep sequencing

## Abstract

**Background:**

Circular RNAs (circRNAs) are a novel group of RNAs and play essential roles in cancers. However, the expression profiles of circRNAs in human colorectal cancer (CRC) are largely unclear.

**Methods:**

The differentially expressed circRNAs, mRNAs, and microRNAs (miRNAs) between CRC tissues and paired adjacent normal tissues were first screened. Then, gene ontology and pathway analyses were performed to predict the possible functions. In addition, we identified the differentially expressed circRNAs in CRC correlated with Krüppel‐like factor 4 (KLF4) and validated their expression levels in CRC tissues. Finally, the correlations between hsa_circ_0142527 expression levels and clinicopathological features of patients with CRC were also analyzed.

**Results:**

After filtered 4735 circRNAs by RNA deep sequencing, 67 differentially expressed circRNAs (fold change >2.0, *P* < 0.05) were selected. The top two pathways were cell cycle and other glycan degradation. Hsa_circ_0142527 and KLF4 mRNA were significantly lower expressed in CRC tissues in both training and confirm groups and have high positive correlation (*r* = 0.754). We further found that the expression levels of hsa_circ_0142527 were significantly associated with age (*P* = 0.004), differentiation (*P* = 0.008), invasion (*P* = 0.029), distal metastasis (*P* = 0.004), TNM stage (*P* = 0.005), and carcinoembryonic antigen (CEA; *P* = 0.037).

**Conclusions:**

The circRNA expression profile of CRC provided new clues for understanding the occurrence of CRC. Hsa_circ_0142527 may be served as a potential biomarker for the diagnosis of CRC.

## 1 INTRODUCTION

Colorectal cancer (CRC) is the third most widespread cancer in men and the second most widespread in women recently. Although screening diagnostic technique like colonoscopy and therapy involved in CRC has made great progress, CRC is fourth in mortality from cancer worldwide.^1^ It is indispensable to find new early diagnostic biomarkers and explore the underlying molecular mechanism of CRC to improve detection rates for early CRC patients and reduced mortality in advanced CRC patients.

Circular RNA (circRNA) is a large number of endogenous RNA in eukaryote, which is a single‐stranded RNA that has a covalent closed loop with no 5′ and 3′ ends.^2^ CircRNAs may arise from exons or introns of pre‐mRNA by back‐splicing.^3^ Interestingly, they play distinctive roles in gene expression regulation and biological processes, participating in serving as microRNA (miRNA) sponge, transcription regulation, rolling circle translation, generating pseudogenes, and affecting alternative splicing.^4^ Recent evidence suggested that circRNAs were closely related to human cancers including CRC mainly through acting as miRNA sponges that can bind with miRNAs to regulate their target genes which having similar miRNA response element (MRE).^3^, ^5^, ^6^

Up to date, RNA deep sequencing, which is gradually replacing in microarrays, has a greater dynamic range compared with microarrays.^7^ In this study, we explored circRNAs in CRC based on RNA deep sequencing and screened differentially expressed circRNAs. Meanwhile, we analyzed the networks among mRNAs, miRNAs, and circRNAs. We performed gene ontology (GO) and pathway analyses of the differentially expressed circRNAs, especially identified them in CRC tissues correlated with Krüppel‐like factor 4 (KLF4), one of CRC‐associated transcription factors. Among them, we randomly selected hsa_circ_0142527 as a targeted circRNA to validate the expression levels in CRC tissues. Its gene is located at chr8_68038292_68022036 with a spliced length of 503 nt. Our results, for the first time, indicated that hsa_circ_0142527 may be served as a potential biomarker for the diagnosis of CRC.

## 2 MATERIALS AND METHODS

### 2.1 Patients and specimens

All specimens were collected from two hospitals in different provinces in China. Forty‐one pairs of CRC tissues and their matched adjacent nontumorous tissues were obtained from surgical patients who were histologically confirmed to have CRC between 2016 and 2017 in the Center Gastroenterology of the Affiliated Hospital of Medical School of Ningbo University (China). The other 50 pairs were gathered from the Department of Abdominal Oncology of Zhejiang cancer hospital between 2010 and 2015. They had not got any therapies before received specimens. At the time of surgery, all tissue specimens were immediately preserved in RNA fixer (Bioteke, Beijing, China) at −80°C until use. Three of them were randomly selected for human RNA deep sequencing. Tumors were classified following the tumor‐node‐metastasis (TNM) staging system (7th ed.). Following the National Comprehensive Cancer Network (NCCN) Clinical Practice Guideline of Oncology (V.1.2012), histologic grade was evaluated. This project was approved by the Human Research Ethics Committee of Ningbo University. All participants received informed consent. Double‐blind manner was used through the overall process.

### 2.2 Library construction and RNA‐seq

Total RNA was extracted from three paired CRC and adjacent normal tissues using TRIzol (Ambion) according to the manufacturer's instructions and was quality controlled using Agilent 2200 (Agilent). Then, total RNA was used for library construction using TruSeq Stranded Total RNA with Ribo‐Zero Gold (Illumina). Following the manufacturer's instructions, strand‐specific RNA‐seq libraries were prepared. The libraries were quality controlled again with Agilent 2200 (Agilent) and sequenced using HiSeq X (Illumina).

### 2.3 RNA sequencing mapping and differentially expressed genes screen

The processed clean reads were then aligned to human genome (version: hg19_GRCh37) using the MapSplice program (v2.1.6).

We filtered the differentially expressed genes using EBSeq algorithm^8^ via the significant analysis including *P*‐value and false discovery rate (FDR) analysis under the following criteria: (a) fold change >1.5 or <0.667 and (b) FDR <0.05.^9^

### 2.4 Go analysis

Gene ontology analysis was performed to facilitate elucidating the biological implications of unique genes in the significant or representative profiles of the differentially expressed gene in the experiment. We downloaded the GO annotations from NCBI (http://www.ncbi.nlm.nih.gov/), UniProt (http://www.uniprot.org/) and the GO (http://www.geneontology.org/). Fisher's exact test was applied to identify the significant GO categories, and FDR was used to correct the *P*‐values.

### 2.5 Pathway analysis

Pathway analysis was used to find out the significant pathway of the differential genes according to Kyoto Encyclopedia of Genes and Genomes (KEGG) database (https://www.kegg.jp/). We turn to the Fisher's exact test to select the significant pathway, and the threshold of significance was defined by *P*‐value and FDR.^10^, ^11^, ^12^

### 2.6 Total RNA extraction, reverse transcription reaction, and qPCR

Tissue RNA was extracted using TRIzol reagents (Ambion), following the manufacturer's instructions, respectively. The A260/A280 ratio in DS‐11 Spectrophotometer (DeNovix) and 1% agarose gel electrophoresis were then used to assess the quality of RNA. Finally, total RNA was reverse transcribed to cDNA by a GoScript Reverse Transcription (RT) System (Promega).

The real‐time quantitative reverse transcription polymerase chain reaction (qRT‐PCR) was performed using GoTaq qPCR Master Mix (Promega) on an Mx3005P Real‐Time PCR System (Stratagene) following the manufacturer's instructions. Divergent primers of hsa_circ_0142527, convergent primers of KLF4, and glyceraldehyde 3‐phosphate dehydrogenase (GAPDH, as a control) were designed and synthesized by Sangon Biotech. The sequences of hsa_circ_0142527, KLF4, and GAPDH were as follows: 5′‐GGGTCCAACATCACTGACACC‐3′ and 5′‐TGTGCCTTCTCATGGTTACTACAA‐3′ for hsa_circ_0142527; 5′‐CCTTCAACCTGGCGGACATCAAC‐3′ and 5′‐GCTGCTGCGGCGGAATGTAC‐3′ for KLF4; and 5′‐ACCCACTCCTCCACCTTTGAC‐3′ and 5′‐TGTTGCTGTAGCCAAATTCGTT‐3′ for GAPDH. The conditions of thermal cycling were as follows: 5 minutes at 95°C for a hot start; then 45 cycles at 94°C for 15 seconds, 54°C for 30 seconds, and 72°C for 30 seconds.^13^ The cycle threshold (Ct) values of hsa_circ_0142527 and GAPDH were recorded. Targeted circRNA (hsa_circ_0142527) expression levels were calculated using the ΔCt method with GAPDH as the control.^14^ Lower ΔCt values mean higher expression levels.

### 2.7 Cloning and sequencing of qRT‐PCR products

The qRT‐PCR products of hsa_circ_0142527 and KLF4 mRNA were purified using the UNIQ‐10 PCR Product Purification Kit (Sangon Biotech) and were cloned into the pUCm‐T vector (Sangon Biotech). Finally, PCR product sequencing was performed by the same company.^15^

### 2.8 Statistical analysis

All statistical analyses are based on Statistical Program for Social Sciences (SPSS) v18.0 (SPSS, lnc.) and GraphPad Prism 5.0 (GraphPad Software, lnc.). Student's *t* test, one‐way analysis of variance (ANOVA), and the rank‐sum test were used according to the actual conditions. The diagnostic value of hsa‐circ‐0142527 was evaluated by receiver operating characteristic (ROC) curve. *P*‐values <0.05 were considered statistically significant.

## 3 RESULTS

### 3.1 Overview of RNA deep sequencing of CRC tissues

Three patient samples were chosen from a CRC cohort. Histopathology was assessed by surgical histopathological examinations, and patient characteristics are shown in Table S1.

**Figure 1:**
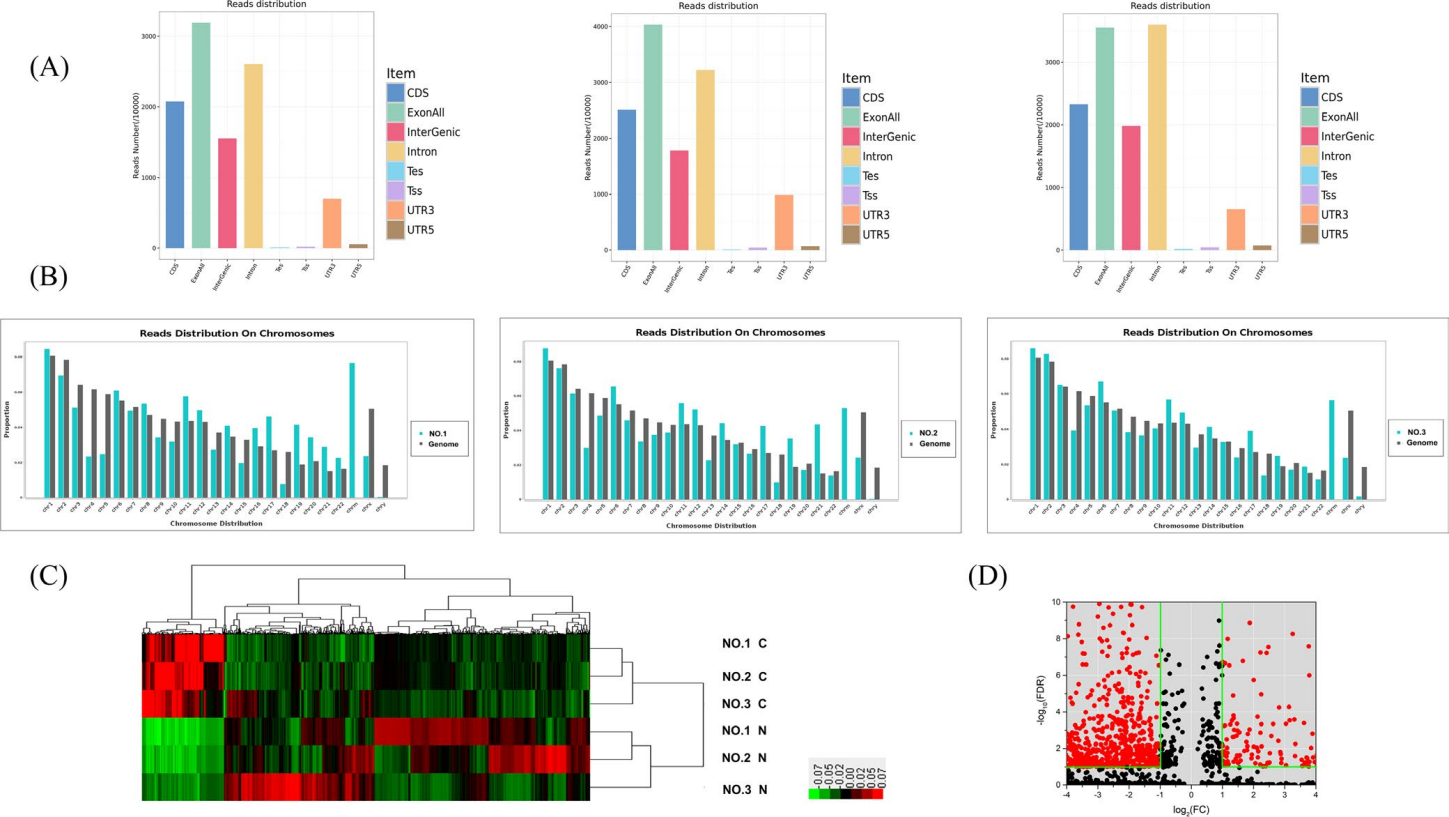
Overview of RNA deep sequencing in colorectal cancer (CRC) tissues. A, All gene structures in transcriptome. B, Chromosomes distribution of transcriptome reads in CRC tissues compared with human genome. C, Hierarchical cluster analysis showed several types of significantly differentially expressed RNAs including mRNAs, noncoding RNAs, and miscellaneous RNAs in CRC and paracancerous tissues. D, The volcano plot. The red dots in the plot represent RNA with statistical significance

After clean reads mapping, a detailed statistical analysis was performed to understand the overview of RNA deep sequencing in CRC tissues (data accessible at NCBI GEO database, accession GSE121842, Zhang https://www.ncbi.nlm.nih.gov/geo/query/acc.cgi?acc=GSE121842). We found that the top three gene structures in transcriptome are exon, intron, and coding sequence (CDS) in CRC (Figure 1A). Besides, we got reads distribution on all chromosomes (Figure 1B).

Hierarchical clustering analysis showed the differential RNA expression levels between CRC and paracancerous tissues (Figure 1C). A volcano plot (Figure 1D) was constructed using fold change (FC) and false discovery rate (FDR) values to visualize the relationship between FC and statistical significance.^16^

### 3.2 Profiles of differentially expressed circRNAs in CRC

**Figure 2:**
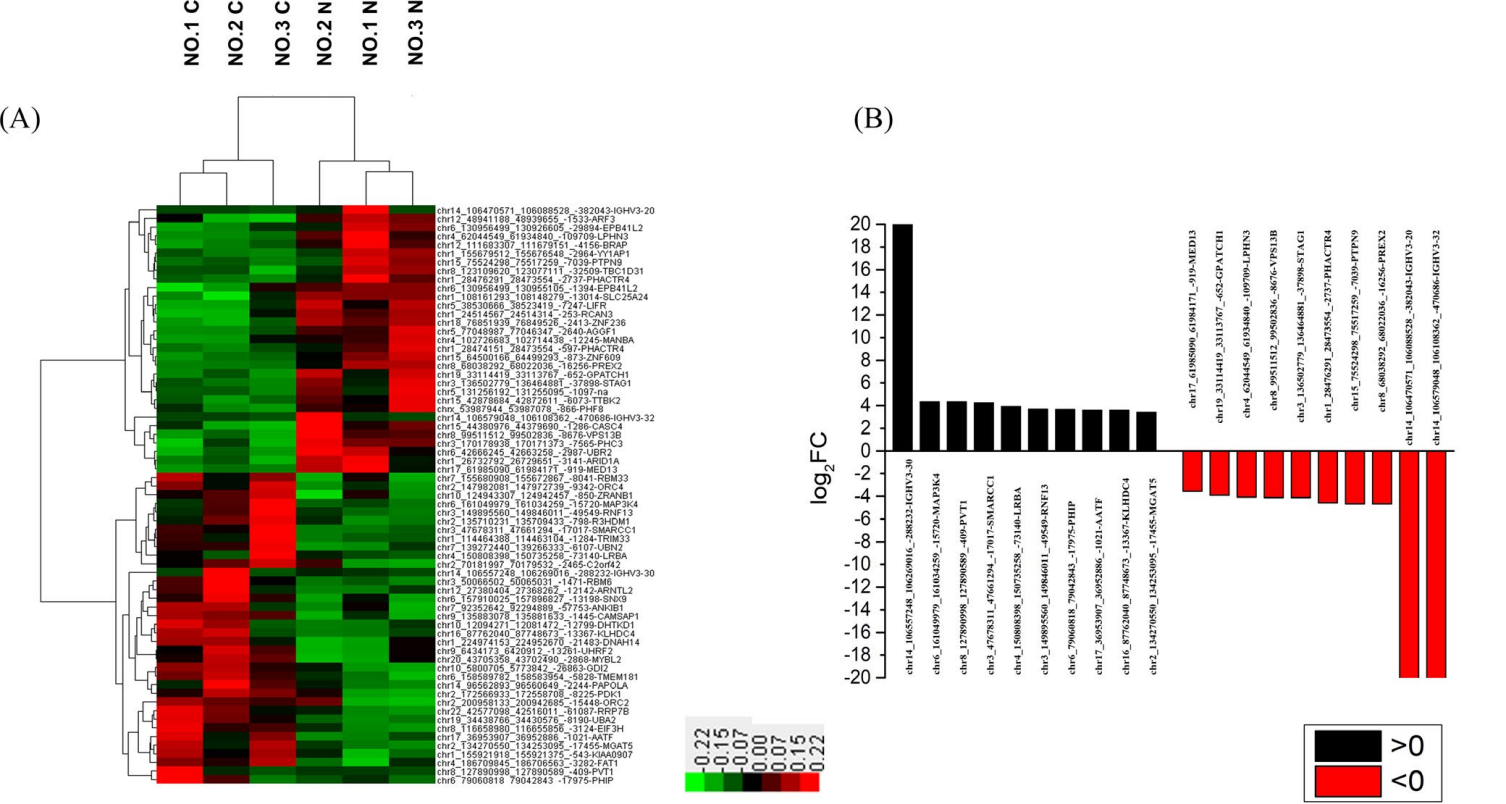
Expression of circRNAs in colorectal cancer (CRC) tissues. A, Hierarchical cluster analysis of differentially expressed circRNAs in CRC and paracancerous tissues (FC >2, FDR <0.05). Significantly differentially expressed circRNAs between normal and malignant tissues of CRC are shown on a scale from green (low) to red (high). Individual tissues subsets are depicted as columns. B, The top 10 upregulated and downregulated circRNAs in CRC tissues

After using EBSeq algorithm, a total of 4735 circRNAs were filtered (GSE121842). Among them, 67 differentially expressed circRNAs were revealed (FC >2.0, FDR <0.05). There are 36 upregulated and 31 downregulated circRNAs, respectively. Hierarchical clustering analysis revealed expression differences of circRNAs between CRC and paracancerous tissues (Figure 2A). According to the log fold change, the top 10 upregulated and downregulated circRNAs in CRC tissue were selected (Table 1, Figure 2B). Among them, six circRNAs did not have circRNA ID in circBase (http://www.circbase.org/).

**Table 1:** The top 10 upregulated and downregulated circRNAs in CRC tissues

CircRNA ID	Chromosome	Regulation	Log^2^FC	FDR	Gene symbol	Strand	NO.	SeqLength
circIGHV3‐30‐1^a^	chr14	Up	20.000	0.000	IGHV3‐30	−	1	288 233
hsa_circ_0078617	chr6	Up	4.322	0.025	MAP3K4	+	2	1555
hsa_circ_0085558	chr8	Up	4.322	0.016	PVT1	+	3	410
hsa_circ_0003602	chr3	Up	4.229	0.008	SMARCC1	−	4	863
hsa_circ_0071174	chr4	Up	3.907	0.034	LRBA	−	5	449
hsa_circ_0067717	chr3	Up	3.672	0.026	RNF13	+	6	425
hsa_circ_0001615	chr6	Up	3.644	0.028	PHIP	−	7	411
circAATF‐1^a^	chr17	Up	3.570	0.005	AATF	+	8	1022
hsa_circ_0000724	chr16	Up	3.570	0.019	KLHDC4	−	9	407
circMGAT5^a^	chr2	Up	3.392	0.011	MGAT5	+	10	17 456
hsa_circ_0002220	chr17	Down	−3.557	0.005	MED13	−	10	503
hsa_circ_0008287	chr19	Down	−3.913	0.007	GPATCH1	+	9	304
hsa_circ_0069865	chr4	Down	−4.068	0.000	LPHN3	+	8	1702
circVPS13‐1^a^	chr8	Down	−4.115	0.004	VPS13B	+	7	666
hsa_circ_0067480	chr3	Down	−4.127	0.009	STAG1	−	6	637
hsa_circ_0000038	chr1	Down	−4.577	0.005	PHACTR4	+	5	783
hsa_circ_0137008/hsa_circ_0142527	chr8	Down	−4.667	0.000	PREX2	+	4	503
hsa_circ_0002578	chr15	Down	−4.667	0.022	PTPN9	−	3	321
circIGHV3‐32‐1^a^	chr14	Down	−20.000	0.000	IGHV3‐32	−	2	470 687
circIGHV3‐20‐1^a^	chr14	Down	−20.000	0.000	IGHV3‐20	−	1	382 044

a No circRNA ID in circBase. We named them after their host gene.

### 3.3 Gene ontology analysis and pathway analysis of differentially expressed circRNAs

Gene ontology analysis of the host genes which producing 67 differentially expressed circRNAs was performed to investigate the potential enriched GO. GO contains three domains: biological process (BP), molecular function (MF), and cellular components (CC).^17^

**Figure 3:**
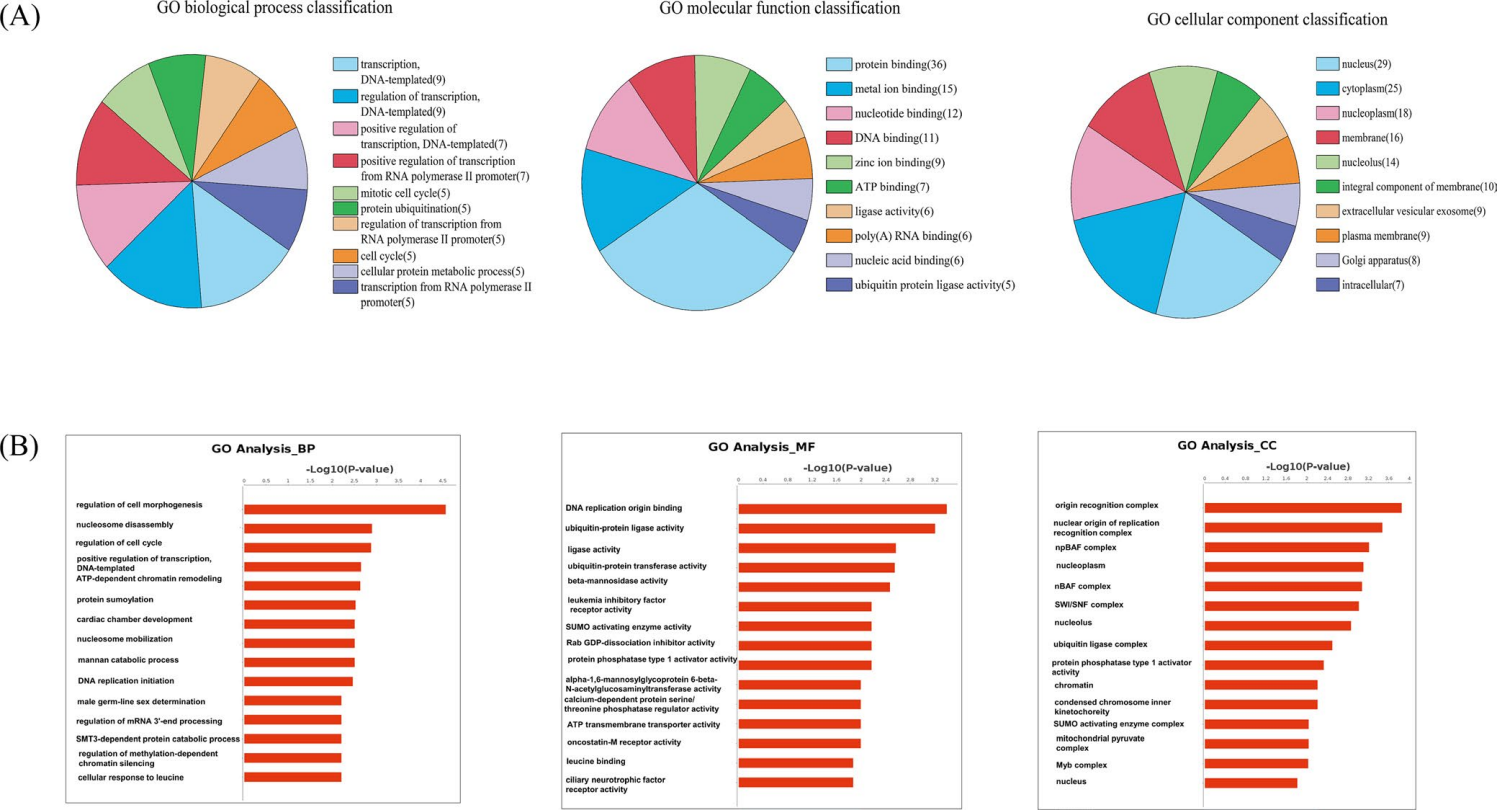
Gene ontology (GO) analysis of the genes which producing 67 differentially expressed circRNAs. A, Top 10 classifications of biological processes (BPs), cellular components (CCs), and molecular functions (MFs) according to the number of linear counterparts of differentially expressed circRNAs. B, The top 15 significant BPs, MFs, and CCs according to the (−log_10_ *P* value) in GO analysis

The enriched GO terms included 265 BPs, 141 MFs, and 93 CCs. According to the number of differential genes in a certain GO ID, the top 10 classifications of BPs, CCs, and MFs were shown (Figure 3A). Among BPs, GO:0006351 (transcription, DNA‐templated) and GO:0006355 (regulation of transcription, DNA‐templated) were the most differential genes. Among MFs, GO:0005515 (protein binding) was the most differential gene. Among CCs, GO:0005634 (nucleus) was the most differential gene. According to the (−log_10_ *P* value), the top 15 BPs, MFs, and CCs were selected (Figure 3B). The top five significant BPs were the regulation of cell morphogenesis, nucleosome disassembly, regulation of cell cycle, positive regulation of transcription, and DNA‐templated. According to the (−log_10_ *P* value) in MFs analysis, the top 15 were selected. The top five significant MFs were DNA replication origin binding, ubiquitin‐protein ligase activity, ligase activity, ubiquitin‐protein transferase activity, and beta‐mannosidase activity. According to the (−log_10_ *P* value), the top 15 CCs were selected. Origin recognition complex, nuclear origin of replication recognition complex, npBAF complex, nucleoplasm, and nBAF complex were top five significant CCs.

**Figure 4:**
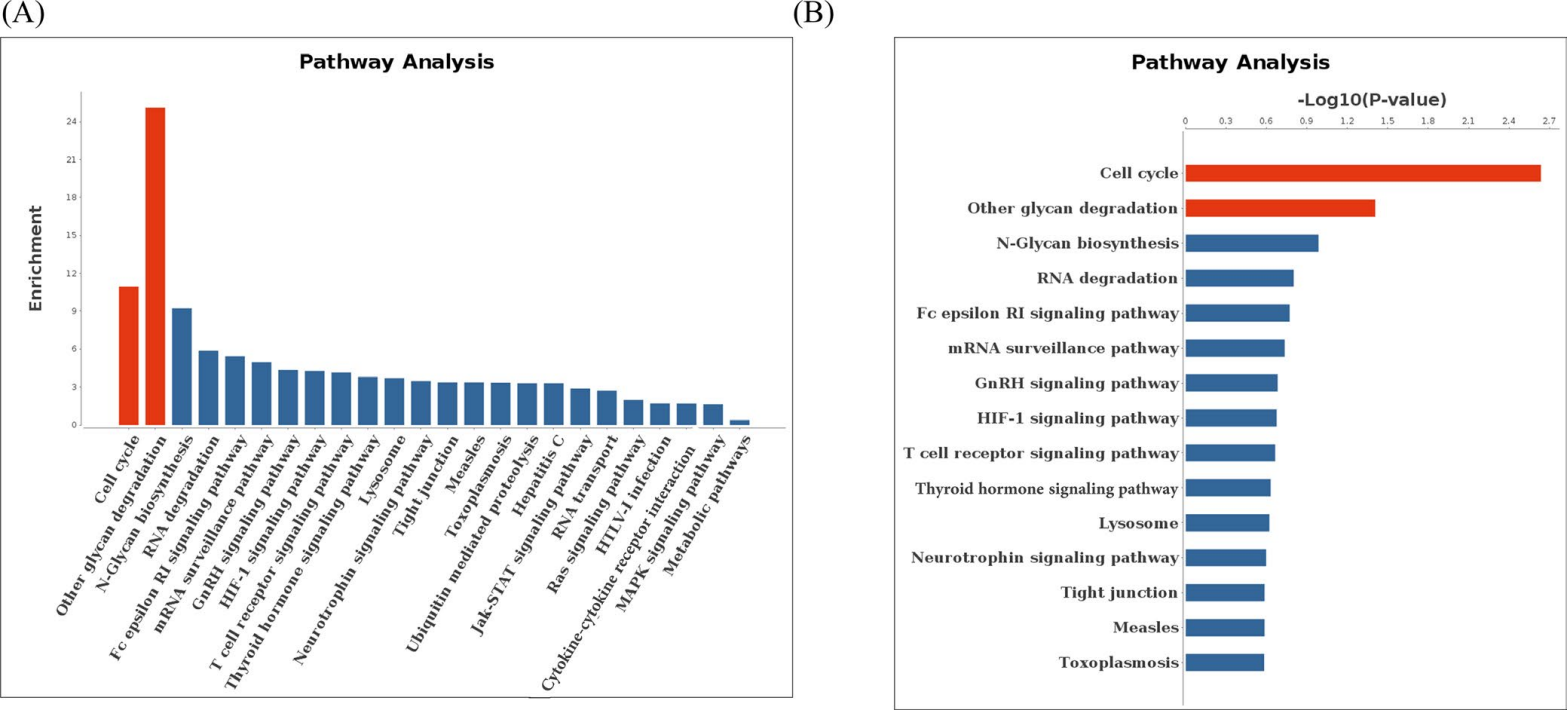
Pathway analysis of the genes which producing 67 differentially expressed circRNAs. A, The arrangement of the host genes producing differentially expressed circRNAs according to the enrichment score. B, The arrangement of the host genes producing differentially expressed circRNAs according to the (−log_10_ *P* value)

To understand the function of differentially expressed circRNAs in CRC tissues, Kyoto Encyclopedia of Genes and Genomes (KEGG) pathway analysis was performed. According to the enrichment score, the top two significant pathways were cell cycle and other glycan degradation (Figure 4A). Interestingly, according to the (−log_10_ *P* value), the top two significant pathways also were cell cycle and other glycan degradation (Figure 4B).

### 3.4 Validation of the expression levels of KLF4 in CRC and identification of differentially expressed circRNAs correlate with KLF4

**Figure 5:**
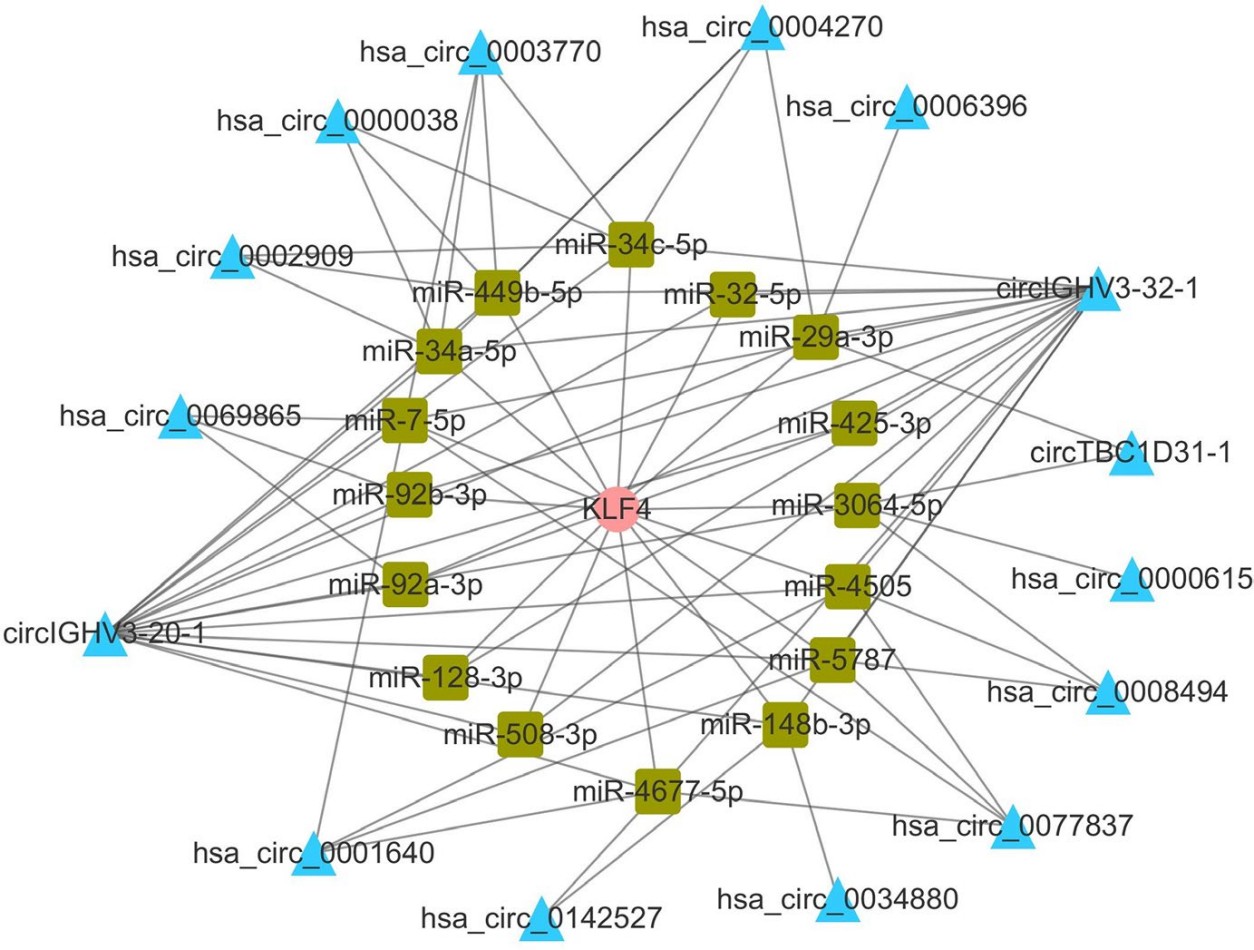
circRNA/miRNA/KLF4 mRNA networks. KLF4 mRNA (pink circle) and 15 circRNAs (blue triangle) shared 16 miRNAs (green square)

Because one of circRNAs' role is acting as miRNA sponges by binding with miRNAs, we predicted circRNAs which have the same binding sites with mRNAs. For example, 15 candidate circRNAs that are KLF4 competing endogenous RNAs (ceRNAs) in CRC tissues were selected (Figure 5).

**Figure 6:**
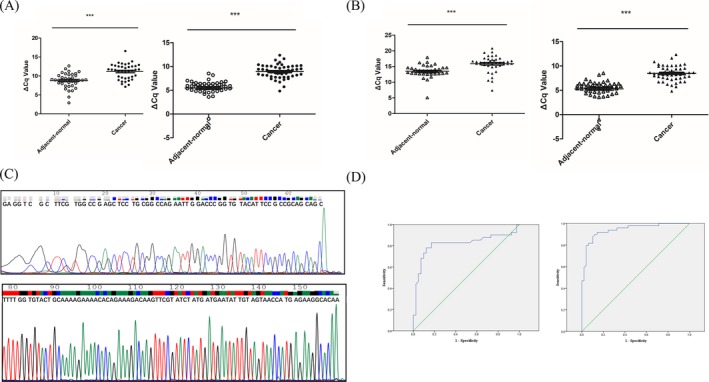
KLF4 mRNA and hsa_circ_0142527 expression levels in paired colorectal cancer (CRC) and paracancerous tissues in training and confirm groups. A, KLF4 expression levels in CRC and paracancerous tissues were detected by qRT‐PCR method. Training group (left): n = 39, ***P < 0.001. Confirm group (right): n = 49, ***P < 0.001. B, hsa_circ_0142527 expression levels in paired CRC and paracancerous tissues were detected by qRT‐PCR method. Training group (left): n = 41, ***P < 0.001. Confirm group (right): n = 49, ***P < 0.001. C, Sequencing results of KLF4 mRNA (top) and hsa_circ_0142527 (below) of qRT‐PCR products. D, The receiver operating characteristic (ROC) curves of hsa_circ_0142527 in polycentricity. Training group (left): the area under the curve (AUC) was 0.818 (95% CI: 0.715‐0.920, *P* < 0.001). Confirm group (right): the AUC was 0.942 (95% CI: 0.896‐0.987, *P* < 0.001)

**Figure 7:**
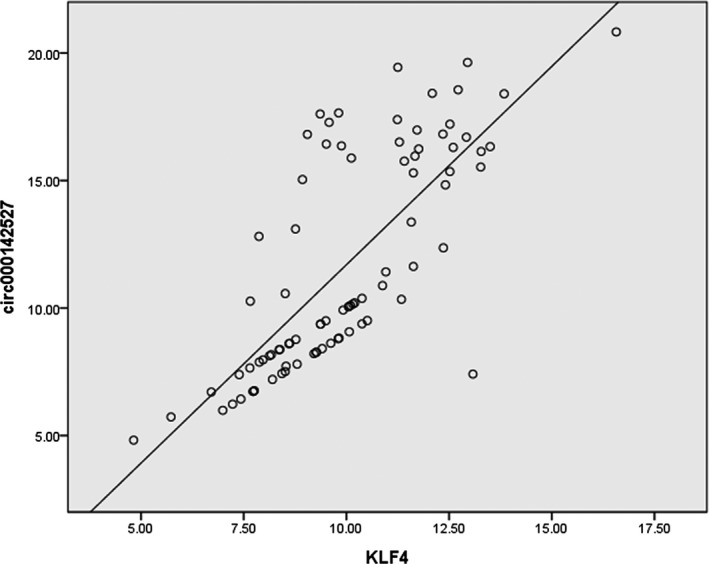
KLF4 and hsa_circ_0142527 have high positive correlation. (*r* = 0.754, *r* ^2^ = 0.568，*P* < 0.001)

Firstly, we verified KLF4 expression levels in paired CRC and paracancerous tissues in polycentricity. The results in both training group and confirm group showed that the expression levels of KLF4 in CRC tissues were significantly lower compared to those in paracancerous tissues (*P* < 0.001, Figure 6A). Then, hsa_circ_0142527 was randomly selected from 15 circRNAs which are KLF4 ceRNAs. The FC and FDR of hsa_circ_0142527 in RNA deep sequencing were −1. 821 and 0.0002, respectively. qRT‐PCR results in polycentricity indicated that hsa_circ_0142527 in CRC tissues was significantly lower compared to paracancerous tissues (*P* < 0.001, Figure 6B). The ROC curve was used for a comprehensive index reflecting the sensitivity and specificity of continuous variable.^18^ The area under the curve (AUC) of hsa_circ_0142527 in training group was 0.818 (95% CI: 0.715‐0.920, *P* < 0.001, Figure 6D). The optimal cut‐off value of hsa_circ_0142527 was 14.625, with sensitivity and specificity 82.9% and 80.5%, respectively. The area under the curve (AUC) of hsa_circ_0142527 in confirm group was 0.942 (95% CI: 0.896‐0.987, *P* < 0.001, Figure 6D). The optimal cut‐off value of hsa_circ_0142527 was 6.72, with sensitivity and specificity 87.8% and 89.8%, respectively. The expression levels of hsa_circ_0142527 and clinicopathological features were also analyzed. We found that the expression levels of hsa_circ_0142527 were significantly associated with age (*P* = 0.004), differentiation (*P* = 0.008), invasion (*P* = 0.029), distal metastasis (*P* = 0.004), TNM stage (*P* = 0.005), and carcinoembryonic antigen (CEA; *P* = 0.037; Table 2). Besides, we sequenced the qRT‐PCR products and confirmed the existence of KLF4 mRNA and hsa_circ_0142527 in CRC tissues (Figure 6C). In addition, we found that KLF4 and hsa_circ_0142527 have high positive correlation (Figure 7; *r* = 0.754, *P* < 0.001).

**Table 2:** The relationships between the expression levels of hsa_circ_0142527 and clinicopathological features of CRC patients

Characteristics	No. of cases (%)	Mean ± SD	*P*‐value
Age (y)			
≥55	65 (72.2)	12.585 ± 4.284	0.004*
<55	25 (27.8)	9.934 ± 3.549	
Gender			
Male	56 (62.2)	12.495 ± 4.236	0.063
Female	34 (37.8)	10.784 ± 4.101	
Tumor location			
Colon	35 (38.9)	12.816 ± 4.347	0.085
Rectum	55 (61.1)	11.233 ± 4.100	
Diameter (cm)			
≥5	36 (40.0)	12.061 ± 4.407	0.701
<5	54 (60.0)	11.707 ± 4.169	
Differentiation			
Well and moderate	77 (85.6)	11.660 ± 4.178	0.008*
Poor	13 (14.4)	8.014 ± 1.940	
Invasion			
T1 & T2 & T3	32 (35.6)	13.176 ± 4.105	0.029*
T4	58 (64.4)	11.117 ± 4.211	
Lymphatic metastasis			
N0	44 (48.9)	12.444 ± 4.376	0.185
N1‐2	46 (51.2)	11.239 ± 4.119	
Distal metastasis			
M0	65 (72.2)	12.627 ± 3.905	0.004*
M1	25 (27.8)	9.824 ± 4.499	
TNM stage			
I & II & III	64 (71.1)	12.620 ± 3.936	0.005*
IV	26 (28.9)	9.824 ± 4.499	
CA19‐9			
Positive	15 (16.7)	10.667 ± 4.433	0.241
Negative	75 (83.3)	12.124 ± 4.201	
CEA			
Positive	29 (32.2)	10.550 ± 4.511	0.037*
Negative	61 (67.8)	12.545 ± 3.989	

* *P* < 0.05.

## 4 DISCUSSION

CircRNAs are a large class of endogenous RNAs in eukaryote. Because of the properties of abundant, stable, diverse, and conserved, circRNAs are involved in various diseases.^19^ Bachmayr‐Heyda et al^20^ firstly reported that a global reduction of circular RNA abundance in CRC cell lines and cancer tissues showed a negative correlation of global circular RNA abundance and proliferation. Zeng et al^12^ showed that CircHIPK3 promoted CRC growth and metastasis by sponging miR‐7. Zeng et al^21^ detected 431 differentially expressed circRNAs from CRC tissues of patients with lung metastasis and constructed circRNA/miRNA interactions, They demonstrated that hsa_circ_102761, hsa_circ_086376, and hsa_circ_105055 bonded with miR‐7 and regulated target genes *EPHA3*, *PRKCB*, *BRCA1*, and *ABCC1* in CRC with lung metastasis.^21^ Besides, Zhang et al^22^ demonstrated that hsa_circ_0020397 regulated CRC cell viability, invasion, and apoptosis by promoting the expression of miR‐138 targets *TERT* and *PD‐L1*. Xie et al^23^ revealed the regulatory mechanism of hsa_circ_001569/miR‐145/*E2F5*, *BAG4*, *FMNL2* axis in cell proliferation and invasion in CRC.

In this study, we performed a human RNA deep sequencing to explore the circRNAs expression profiles in CRC (Figure 1). We found 67 differentially expressed circRNAs, 36 upregulated and 31 downregulated circRNAs, respectively (Figure 2). Among the top 10 upregulated and downregulated circRNAs in CRC, six circRNAs had no circRNA ID in circBase (Table 1). These circRNAs may be new circRNAs. Besides, all the top 10 upregulated and 10 downregulated circRNAs were not found in CRC cancer tissues before October 24, 2018, in the literatures from PubMed (https://www.ncbi.nlm.nih.gov/pubmed/). These results provide new information for further understanding the occurrence of CRC.

One major mechanism of circRNAs is acting as miRNAs to regulate gene expression. It is now well established that KLF4 (also called gut‐enriched Krüppel‐like factor or GKL)^24^ is a key suppressor gene in CRC involved in multiple key CRC pathways such as Notch pathway and Wnt/β‐catenin pathway.^25^, ^26^, ^27^, ^28^, ^29^ A number of miRNAs can bind to the 3′‐untranslated region (3′‐UTR) of KLF4 mRNA.^25^, ^26^, ^27^, ^30^, ^31^, ^32^, ^33^, ^34^ Hence, we predicted 15 candidate circRNAs as KLF4 ceRNA in CRC tissues (Figure 5). Hsa_circ_0142527 was selected as a targeted circRNA to validate the accuracy of RNA deep sequencing. Our results showed that hsa_circ_0142527 in CRC was significantly lower compared to matched paracancerous tissues (Figure 6). The area under the ROC curves was 0.702 (Figure 6D). More importantly, the expression levels of hsa_circ_0142527 were significantly associated with age, differentiation, invasion, distal metastasis, TNM stage, and carcinoembryonic antigen (CEA; Table 2). The moderate correlations between KLF4 and hsa_circ_0142527 were documented (Figure 7).

Nowadays, whether circRNAs can regulate host genes is controversial. Du et al^35^ found that over‐expressed circ‐DNMT1 increased the mRNA level of the parent gene *DNMT1*. A recent study indicated that circITGA7 inhibited CRC growth and metastasis by modulating Ras pathway and upregulating transcription of its host gene *ITGA7*.^36^ Besides, circEIF3J and circPAIP2 showed interactions with RNA Pol II, U1 snRNP, and parental gene promoters to promote the transcription of host genes.^37^ These results indicate that some circRNAs can regulate their host genes.

Furthermore, we performed GO analysis of the host genes producing the differentially expressed circRNAs. The top five significant BPs were regulation of cell morphogenesis, nucleosome disassembly, regulation of cell cycle, positive regulation of transcription, and DNA‐templated (Figure 3). It was required for transcription controls that underlie differentiation, development, and tumor suppression.^38^ Another previous study also revealed apoptosis‐antagonizing transcription factor (*AATF*), one number of differentially expressed circRNA circAATF‐1, was involved in transcriptional regulation, cell cycle control, DNA damage responses, and in the execution of cell death programs.^39^ GO analysis showed that the occurrence and progress of CRC were co‐regulated by differentially expressed circRNAs from different functional systems (Figure 3). Moreover, according to the enrichment score or (−log_10_ *P* value), the top 2 pathways were cell cycle and other glycan degradation (Figure 4). In our study, three differentially expressed circRNAs (ORC4, STAG1, and ORC2) were predicted involved in cell cycle.

In conclusion, our study provided a circRNA expression profiles in CRC and established a network among miRNAs, mRNAs, and circRNAs in CRC. We also firstly demonstrated that hsa_circ_0142527 may be a potential new biomarker for the diagnosis of CRC.
