# Supplementary material for: Expression profiles of circular RNAs in human colorectal cancer based on RNA deep sequencing
Source: J Clin Lab Anal. 2019 Jun 6;33(7):e22952. doi: 10.1002/jcla.22952 (PMC6757124; doi:10.1002/jcla.22952)
Supplement: Supplementary file 1 [file JCLA-33-e22952-s001.docx]

Supplementary Table 1. Clinical and histopathological characteristics of patients for RNA deep sequencing in this study

| Patient No. | Age  (years) | Gender | Histology | Differentiation | TNM  classification | Anatomic  site |
| --- | --- | --- | --- | --- | --- | --- |
| 1 | 54 | female | Adenocarcinoma | moderate | T3N1M0 (ⅢB) | rectum |
| 2 | 70 | female | Adenocarcinoma | low | T3N1M0 (ⅢB) | colon |
| 3 | 49 | male | Adenocarcinoma | low | T4N1bM0 (ⅢB) | rectum |

TNM：tumor-node-metastasis
